# Efficient Combination Chemo-Sonodynamic Cancer Therapy Using Mitochondria-Targeting Sonosensitizer-Loaded Polysorbate-Based Micelles

**DOI:** 10.3390/ijms25063474

**Published:** 2024-03-20

**Authors:** Hyeon Ju Kang, Quan Truong Hoang, Jun Min, Min Soo Son, Le Thi Hong Tram, Byoung Choul Kim, Youngjun Song, Min Suk Shim

**Affiliations:** Department of Nano-Bioengineering, Incheon National University, Incheon 22012, Republic of Korea; yuj005129@inu.ac.kr (H.J.K.); lthtram200197@gmail.com (L.T.H.T.);

**Keywords:** sonodynamic therapy, polysorbate, Tween 80, mitochondria, piperlongumine

## Abstract

Sonodynamic therapy (SDT), utilizing ultrasound (US) and sonosensitizers, holds immense potential as a noninvasive and targeted treatment for a variety of deep-seated tumors. However, the clinical translation of SDT is hampered by several key limitations in sonosensitizers, especially their low aqueous stability and poor cellular uptake. In this study, non-ionic polysorbate (Tween 80, T80) was adopted to formulate effective nanocarriers for the safe and efficient delivery of sonosensitizers to cancer cells. Mitochondria-targeting triphenylphosphonium (TPP)-conjugated chlorin e6 (Ce6) sonosensitizer was loaded into T80-based micelles for efficient SDT. Pro-oxidant piperlongumine (PL) was co-encapsulated with TPP-conjugated Ce6 (T-Ce6) in T80 micelles to enable combination chemo-SDT. T80 micelles substantially enhanced the cellular internalization of T-Ce6. As a result, T80 micelles loaded with T-Ce6 and PL [T80(T-Ce6/PL)] significantly elevated intracellular reactive oxygen species (ROS) generation in MCF-7 human breast cancer cells upon US exposure. Moreover, T-Ce6 exhibited selective accumulation within the mitochondria, leading to efficient cell death under US irradiation. Importantly, T80(T-Ce6/PL) micelles caused cancer-specific cell death by selectively triggering apoptosis in cancer cells through PL. This study demonstrated the feasibility of using T80(T-Ce6/PL) micelles for efficient and cancer-specific combination chemo-SDT.

## 1. Introduction

Sonodynamic therapy (SDT) represents a non-invasive strategy for deep tumor treatment, where focused ultrasound (US) activates sonosensitizers to produce reactive oxygen species (ROS) for localized cell damage [1,2,3]. SDT leverages the superior tissue penetration of US compared to light in photodynamic therapy (PDT), enabling the treatment of deep-seated tumors while avoiding damage to healthy tissues nearby [4,5]. Despite the promising features of SDT, its anticancer activity is limited by the ineffective intracellular delivery of sonosensitizers [5]. Therefore, it is crucial to develop safe drug carriers capable of augmenting the cellular uptake and bioavailability of sonosensitizers to ensure effective SDT [6].

Recently, subcellular organelle-targeting cancer therapy has gained great attention for effective and specific cancer treatment [7,8,9]. Mitochondria play a crucial role in cellular physiological processes such as apoptosis regulation and calcium homeostasis [8]. They are also mainly responsible for cellular energy production [9]. Therefore, targeting mitochondrial function emerges as a promising strategy for efficient cancer therapy, as it can trigger cell death, inhibit ATP production, and potentially overcome drug resistance in cancer cells [10]. Additionally, mitochondria are highly susceptible to oxidative damage caused by an increase in intracellular ROS levels [11]. Therefore, utilizing mitochondria-targeting sonosensitizers can be a strategic approach to increase the therapeutic efficacy of SDT [12]. 

Nanocarrier-mediated delivery of sonosensitizers has emerged as a potent approach to overcome the significant limitations of sonosensitizers [13,14,15]. Nanocarriers play a crucial role in enhancing the cellular internalization and bioavailability of sonosensitizers [16,17]. Recently, polysorbates such as Tween 80 (T80), a class of non-ionic surfactants, have been widely employed to formulate effective nanocarriers for the safe and efficient delivery of therapeutic agents [18,19,20]. Beyond the low toxicity and minimal hemolysis of polysorbates, their lipophilic nature enables the encapsulation of both hydrophilic and hydrophobic drugs [20]. 

Recently, pro-oxidant cancer therapy, employing drugs that induce cytotoxic oxidative stress, has emerged as a promising strategy for cancer-selective treatment [21,22,23,24]. Piperlongumine (PL) is a new class of pro-oxidant agents and has exhibited selective cytotoxicity towards cancer cells [21,24]. While PL holds therapeutic promise, its limited water solubility hampers clinical applications, restricting administration routes and reducing bioavailability [25,26]. Therefore, the development of drug carriers capable of encapsulating hydrophobic PL is crucial for enhancing its in vivo anticancer efficacy. 

**Figure 1:**
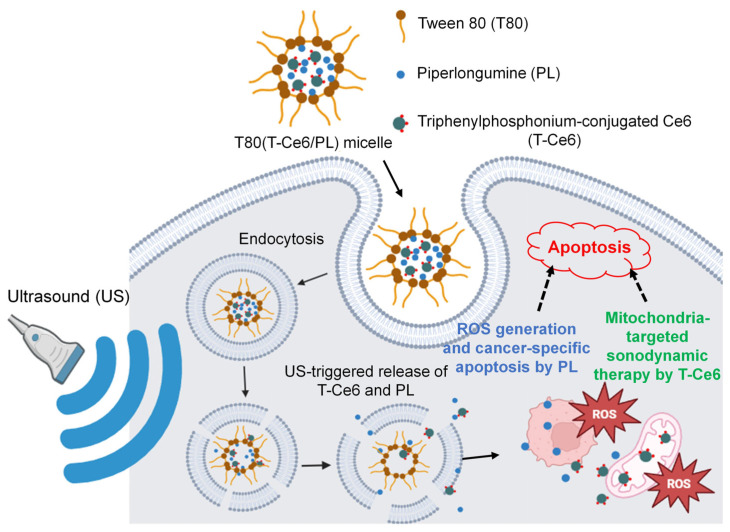
Schematic representation of T80(T-Ce6/PL) micelle-based combination chemo-sonodynamic cancer therapy. T-Ce6 and PL are released from T80(T-Ce6/PL) micelles once they enter the breast cancer cells through endocytosis. Under US irradiation, the released T-Ce6 effectively accumulates in the mitochondria and produces ROS. The ROS triggers the rupture of mitochondria and thus induces apoptosis. Simultaneously, the released PL triggers cancer-specific apoptosis by excessive ROS production, enabling combination chemo-SDT.

In this study, T80-based micelles encapsulating sonosensitizers and pro-oxidant chemodrugs were developed for combination chemo-SDT (Figure 1). Mitochondria-targeting triphenylphosphonium (TPP) [12,27] was conjugated to chlorin e6 (Ce6) sonosensitizer for enhanced accumulation within mitochondria, leading to mitochondria-targeted SDT. Pro-oxidant PL was co-encapsulated with T-Ce6 in T80-based micelles. Under US irradiation, the encapsulated T-Ce6 triggers mitochondria-targeted SDT, producing ROS for targeted mitochondrial damage. Co-delivered PL further enhances the generation of ROS, potentiating the synergistic antitumor effect. We assessed the physicochemical properties of T-Ce6- and PL-loaded T80-based micelles [T80(T-Ce6/PL)]. Furthermore, we assessed the combined therapeutic effects of the micelles through in vitro studies.

## 2. Results and Discussions 

### 2.1. Characterization of T80(T-Ce6/PL) Micelles

**Figure 2:**
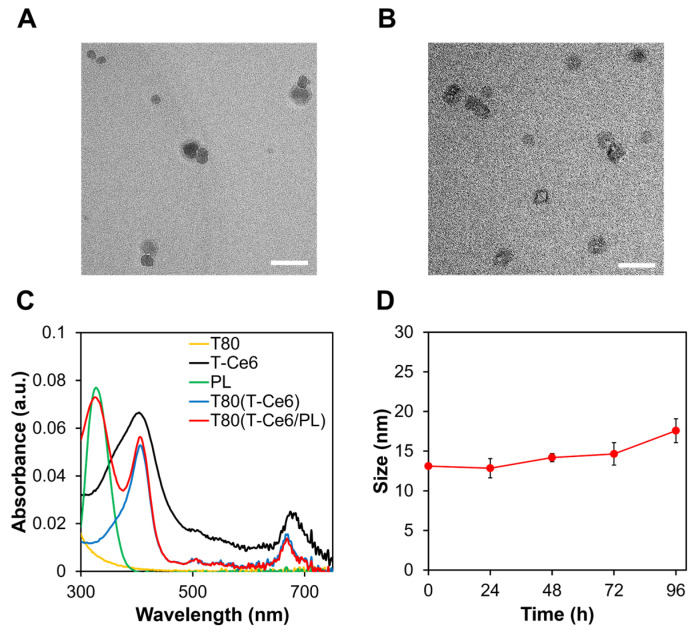
TEM images of (**A**) T80(T-Ce6) and (**B**) T80(T-Ce6/PL). Scale bars: 50 nm. (**C**) Absorbance spectra of blank T80, T-Ce6, PL, T80(T-Ce6), and T80(T-Ce6/PL). (**D**) Size changes of T80(T-Ce6/PL) after incubation in 10% FBS/PBS for various periods of time. (*n* = 3).

It is well-known that polysorbates form micelles in water due to their amphiphilic nature [28]. Hydrophobic drugs can be entrapped into the hydrophobic core of polysorbate micelles. The sizes and zeta potentials of blank T80 and T80(T-Ce6/PL) micelles were measured (Table 1). The sizes of blank T80, T80(T-Ce6), and T80(T-Ce6/PL) were 10.48 ± 0.46, 18.03 ± 0.36, and 18.28 ± 8.49 nm, respectively. The size of T80-based micelles increased when loaded with T-Ce6 and PL. The zeta potentials of blank T80, T80(T-Ce6), and T80(T-Ce6/PL) micelles were −18.4 ± 7.80, −9.36 ± 0.75, and −9.08 ± 4.64 mV, respectively. The size and morphology of T80(T-Ce6) and T80(T-Ce6/PL) micelles were visualized using transmission electron microscopy (TEM). TEM images revealed that T80(T-Ce6) and T80(T-Ce6/PL) micelles exhibited a spherical morphology, with diameters ranging from 15 to 25 nm (Figure 2A,B). The nanoscale T80-based micelles hold the potential for targeted drug delivery to tumors by leveraging the enhanced permeability and retention (EPR) effect [29,30]. The effective encapsulation of T-Ce6 and PL into T80-based micelles was verified by UV-Vis spectroscopy. The absorption spectra of T80, free PL, free T-Ce6, and T80(T-Ce6/PL) are displayed in Figure 2C. T80(T-Ce6/PL) micelles displayed a characteristic absorption peak of T-Ce6 at 660 nm. In addition, we observed the distinctive absorption peak of PL at 328 nm. The absorption peaks indicated that both T-Ce6 and PL were successfully encapsulated in T80-based micelles. We calculated the encapsulation efficiencies of T-Ce6 and PL in T80(T-Ce6/PL) micelles using UV-Vis spectroscopy, revealing the values of approximately 70.6% for T-Ce6 and 35.8% for PL.

### 2.2. High Colloidal Stability of T80(T-Ce6/PL) under Physiological Conditions

To assess the colloidal stability of T80(T-Ce6/PL) micelles against serum proteins, T80(T-Ce6/PL) was suspended in phosphate-buffered saline (PBS) with 10% (*v*/*v*) fetal bovine serum (FBS) at 37 °C. To examine whether there was any aggregation during incubation, the size change of the T80(T-Ce6/PL) was monitored. T80(T-Ce6/PL) showed a slight increase in size after incubation with serum proteins for 4 days (Figure 2D). The minimal aggregation of T80(T-Ce6/PL) micelles, even in the presence of serum proteins, demonstrates their robust stability in biologically relevant environments. Therefore, T80(T-Ce6/PL) micelles would circulate in the bloodstream for an extended period, allowing them to reach tumors more effectively, potentially improving treatment outcomes.

### 2.3. US-Triggered Drug Release from T80(T-Ce6/PL)

**Figure 3:**
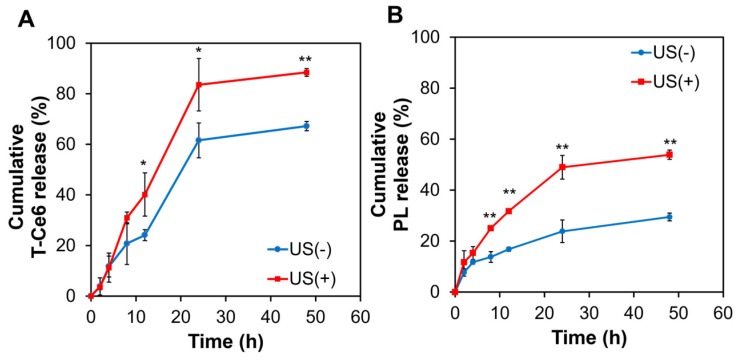
(**A**) T-Ce6 and (**B**) PL released from T80(T-Ce6/PL) in PBS (pH 7.4, 37 °C) before and after US treatment (1 MHz, 0.3 W/cm^2^, 2 min). (*n* = 3, * *p* < 0.05, ** *p* < 0.01).

The release profiles of T-Ce6 and PL from T80(T-Ce6/PL) were assessed under physiological environments (37 °C, pH 7.4). We measured the cumulative amounts of PL and T-Ce6 released from T80(T-Ce6/PL) before and after exposure to US in order to assess the drug release patterns of T80(T-Ce6/PL). The drug release pattern of T80(T-Ce6/PL) micelles exhibited an incubation time-dependent behavior, regardless of US irradiation (Figure 3A,B). The cumulative amount of T-Ce6 and PL increased substantially when T80(T-Ce6/PL) was pretreated with US (Figure 3A,B). US irradiation (2 min) significantly boosted T-Ce6 release from T80(T-Ce6/PL) micelles, achieving 83.5% release within 24 h, compared to only 61.6% without US treatment. US irradiation also facilitated the release of PL. This result demonstrates the US-triggered drug release from T80-based micelles, offering remarkable control over drug release kinetics.

### 2.4. Enhanced Cellular Uptake of T80(T-Ce6/PL)

**Figure 4:**
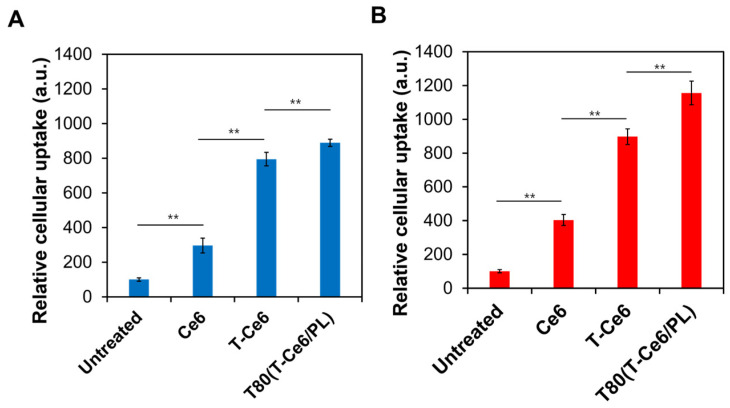
Relative cellular uptake of Ce6, T-Ce6, and T80(T-Ce6/PL) in (**A**) hDFB and (**B**) MCF-7 cells (*n* = 3, ** *p* < 0.01).

We examined the cellular internalization levels of Ce6, T-Ce6, and T80(T-Ce6) micelles in MCF-7 human breast cancer cells and human dermal fibroblast cells (hDFB). hDFB cells were used as non-cancerous cells. As illustrated in Figure 4A,B, irrespective of the cell line, the cellular internalization levels of T80(T-Ce6/PL) were much higher than those of free Ce6 and free T-Ce6. The enhanced cellular uptake of T-Ce6 by T80-based micelles might be explained by the high affinity between T80 and cell membranes, as proposed by previous work [15]. This result suggests that T80-based micelles are effective carriers for enhanced cellular uptake of T-Ce6. During the US irradiation, the cavitation effect of US may facilitate the cellular uptake of T-Ce6 by inducing the disruption of cell membranes [31]. This may increase the internalization of T-Ce6 by the cells. 

### 2.5. Enhanced Mitochondrial Uptake of T-Ce6

**Figure 5:**
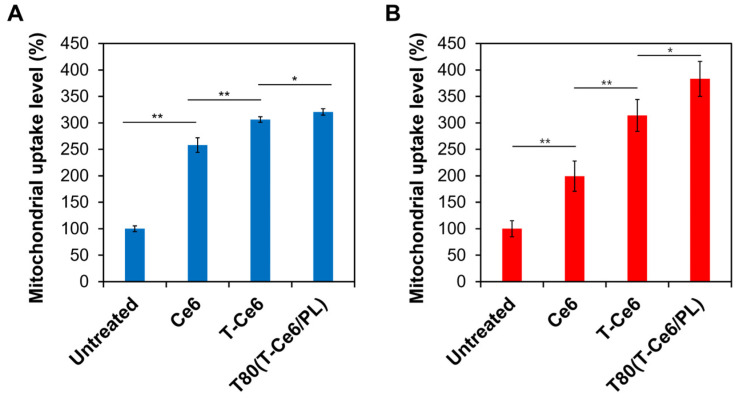
Relative mitochondrial uptake degrees of Ce6, T-Ce6, and T80(T-Ce6/PL) in (**A**) hDFB and (**B**) MCF-7 cells (*n* = 3, * *p* < 0.05, ** *p* < 0.01).

Ce6 was linked to mitochondria-targeting TPP to augment its mitochondrial localization. To investigate the mitochondria-targeting ability of T-Ce6, the amounts of T-Ce6 and Ce6 localized into the mitochondria were measured in both MCF-7 and hDFB cells. The mitochondrial targeting efficiency of T-Ce6 was determined by calculating the ratio of the amount of T-Ce6 that localized in the mitochondria to the total amount of the drug that entered the cell. As shown in Figure 5A,B, the mitochondria uptake of T-Ce6 was significantly higher than Ce6, irrespective of the cell line. When T-Ce6 was loaded into T80, the mitochondrial uptake of T-Ce6 increased compared to the other samples. It is hypothesized that US can increase the mitochondrial uptake of T-Ce6. As mitochondria are highly susceptible to ROS, ROS generated from T-Ce6 under US irradiation can induce the destruction of mitochondrial membranes [32]. The increased permeability of the mitochondrial membranes may facilitate a more efficient accumulation of T-Ce6 within the mitochondria. 

**Figure 6:**
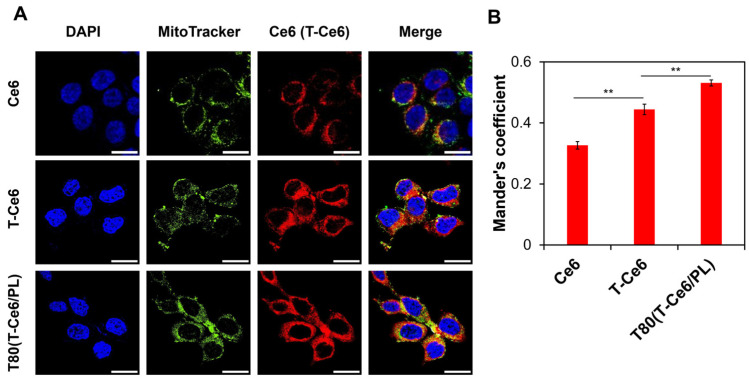
(**A**) Confocal micrographs representing the mitochondrial distribution of Ce6, T-Ce6, and T80(T-Ce6/PL) in MCF-7 cells. Scale bars: 25 µm. (**B**) Mander’s overlap coefficients determined by the co-localization between red fluorescence (from Ce6 or T-Ce6) and green fluorescence (from MitoTracker Green) (*n* = 3, ** *p* < 0.01).

Confocal laser scanning microscopy (CLSM) was adopted to provide additional evidence for the mitochondrial accumulation of T-Ce6 in cancer cells. As shown in Figure 6A, the red fluorescence signals of Ce6 or TPP-Ce6 substantially overlapped with the green fluorescence of mitochondria labeled with MitoTracker Green. To measure the level of T-Ce6 (or Ce6) colocalization in the mitochondria quantitatively, we employed Mander’s coefficients, which analyze the overlap between red (T-Ce6/Ce6) and green (mitochondrial marker) fluorescence signals. As shown in Figure 6B, T-Ce6 exhibited significantly higher mitochondrial colocalization compared to Ce6. Moreover, T80(T-Ce6/PL) group exhibited a higher Mander’s overlap coefficient than T-Ce6 group (0.531 for T80(T-Ce6/PL) and 0.444 for T-Ce6). These findings provide strong evidence that T-Ce6 is effective in mitochondria targeting, mainly due to the mitochondria-targeting characteristics of TPP.

### 2.6. ROS Generation of T80(T-Ce6/PL) upon US Exposure

**Figure 7:**
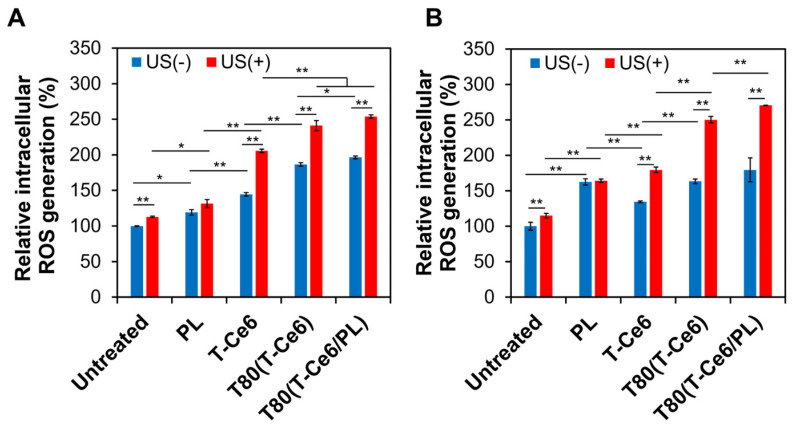
Relative intracellular ROS levels in (**A**) hDFB and (**B**) MCF-7 cells that received PL, T-Ce6, T80(T-Ce6), and T80(T-Ce6/PL) [5 µM T-Ce6, 8 µM PL, 30 µg/mL T80] before and after US irradiation (1 MHz, 0.3 W/cm^2^, 2 min) (*n* = 3, * *p* < 0.05, ** *p* < 0.01).

To demonstrate the efficacy of T-Ce6 in generating ROS under US irradiation, we measured the level of intracellular ROS in hDFB and MCF-7 cells following the incubation with T-Ce6 and T-Ce6-loaded micelles before and after US irradiation. Both hDFB and MCF-7 cells increased intracellular ROS levels after treatment with PL, regardless of US irradiation (Figure 7A,B). Notably, MCF-7 cells treated with PL exhibited a higher increase in intracellular ROS levels compared to hDFB cells. This finding is consistent with our earlier observation that PL treatment selectively enhances ROS levels in cancer cells as opposed to normal cells [10]. Regardless of the US treatment and cell line, T80(T-Ce6) treatment resulted in elevated concentrations of intracellular ROS compared to free T-Ce6 treatment (Figure 7A,B). This result might be ascribed to the enhanced cellular uptake of T-Ce6 when encapsulated in T80 micelles. As displayed in Figure 7A,B, US exposure significantly amplified ROS generation by T-Ce6, T80(T-Ce6), and T80(T-Ce6/PL) in both cell lines. Notably, the highest level of intracellular ROS generation was achieved by the treatment with T80(T-Ce6/PL) and US for both hDFB and MCF-7 cells. This finding demonstrates the synergistic action of sonodynamic effects by T-Ce6 and the pro-oxidant effect of PL.

### 2.7. Cancer-Specific Chemo-SDT Using T80(T-Ce6/PL)

**Figure 8:**
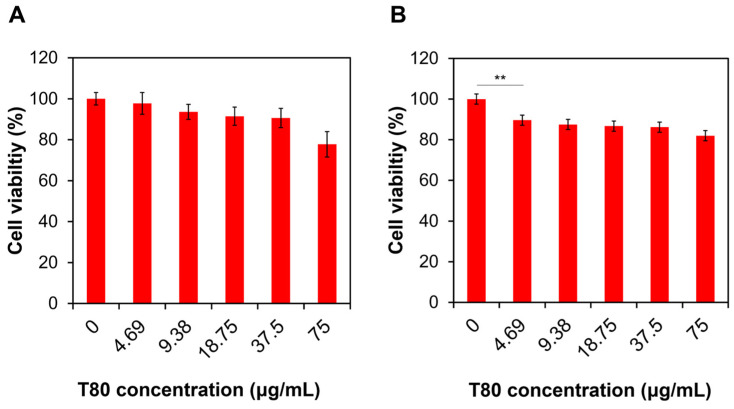
The viability of (**A**) hDFB and (**B**) MCF-7 cells treated with blank T80 at various concentrations (*n* = 3, ** *p* < 0.01).

The US-triggered cytotoxic effects of T-Ce6-loaded T80 micelles were evaluated in MCF-7 cells to demonstrate their potential for chemo-sonodynamic combination therapy. To further demonstrate the cancer-specific cytotoxicity of T80(T-Ce6/PL) micelles, their cytotoxicity was evaluated and compared in MCF-7 and hDFB cells. To demonstrate the biocompatibility of T80-based micelles, the viability of MCF-7 and hDFB cells was measured after incubation with blank T80-based micelles at various concentrations. Both MCF-7 and hDFB cells remained at least 75% viable at concentrations up to 75 µg/mL of T80 (Figure 8). The low cytotoxicity of T80 micelles suggests that they are safe nanoplatforms for drug delivery. 

**Figure 9:**
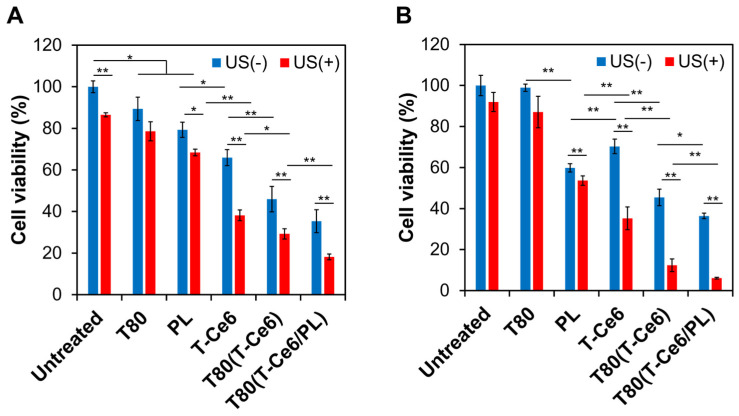
The viability of (**A**) hDFB and (**B**) MCF-7 cells treated with blank T80, PL, T-Ce6, T80(T-Ce6), and T80(T-Ce6/PL) [5 µM T-Ce6, 8 µM PL, 30 µg/mL T80] before and after 2 min of US irradiation (1 MHz, 0.3 W/cm^2^) (*n* = 3, * *p* < 0.05, ** *p* < 0.01).

As shown in Figure 9A,B, US exposure (0.3 W/cm^2^, 2 min, 1 MHz) alone exhibited minimal cytotoxicity (cell viability higher than 85% for both MCF-7 and hDFB cells). When the cells were irradiated with US for 2 min, the temperature increased by approximately 4 °C. This minimal temperature increase of 4 °C is not anticipated to have a substantial impact on the viability of the cells [18]. Additionally, blank T80 micelles (35 µg/mL) did not cause significant toxicity against hDFB and MCF-7 cells (viability > 78%). The low cytotoxicity of T80 micelles suggests that they are safe nanoplatforms for SDT. Notably, PL induced higher cytotoxic effects in MCF-7 cells than in hDFB cells, regardless of US treatment (Figure 9A,B), indicating PL-triggered cancer-specific cytotoxicity. Treatment with T-Ce6, T80(T-Ce6/PL), and T80(T-Ce6/PL) at the concentration of 5 μM T-Ce6 and 10 µM PL resulted in a significant reduction in the cell viability for both MCF-7 and hDFB cells when exposed to US. This result can be explained by the T-Ce6-induced SDT effect. The combination of T80(T-Ce6/PL) and US exhibited the most substantial cytotoxicity in both hDFB and MCF-7 cells. Moreover, T80(T-Ce6/PL) combined with US irradiation reduced the viability of MCF-7 cells more significantly than that of hDFB cells. This result demonstrates that T-Ce6 and PL synergistically enhance anticancer effects through mitochondria-targeted SDT and tumor-specific chemotherapy, respectively, offering a promising therapeutic strategy. This synergistic approach not only amplifies anticancer effects but also potentially reduces side effects through US-triggered, cancer-specific cytotoxicity.

## 3. Materials and Methods

### 3.1. Materials

2′,7′-Dichlorofluorescein diacetate (DCF-DA) and T80 were supplied from Sigma Aldrich (St. Louis, MO, USA). PL was purchased from Cayman Chemical (Ann Arbor, MI, USA). Ce6 was supplied from Hangzhou Dayangchem Co., Ltd. (Hangzhou, China). FBS, cell culture media, and penicillin-streptomycin were purchased from Hyclone (Logan, UT, USA). MitoTracker Green was supplied from Thermo Fisher Scientific (Waltham, MA, USA). US treatment was conducted using a Sonicator 740 (Mettler Electronics Corp., Anaheim, CA, USA). MCF-7 and hDFB cells were obtained from ATCC (Manassas, VA, USA). Fetal bovine serum (FBS) and penicillin-streptomycin were purchased from Hyclone (Cytiva, Marlborough, MA, USA). Minimum essential medium/Earle’s balance salt solutions (MEM/EBSS) and Dulbecco’s modified Eagle medium (DMEM) (Cytiva, Marlborough, MA, USA) were used for the culture of MCF-7 and hDFB cells, respectively.

### 3.2. Preparation and Characterization of T80-Based Micelles Encapsulating T-Ce6 and PL

Mitochondria-targeting T-Ce6 was synthesized as previously reported [33]. To prepare T-Ce6-loaded T80 micelles, 0.3 µM of T-Ce6 in 30 µL of DMSO was prepared and incubated with of 3% (*w*/*v*) T80 in PBS (1 mL). In addition, to prepare T-Ce6- and PL-loaded T80 micelles, 0.3 µM of T-Ce6 (30 µL) and 0.6 µM of PL (60 µL) in DMSO solution were prepared and incubated with 1 mL of 3% (*w*/*v*) T80 in PBS. The mixtures were stirred at 600 rpm for 1 h at 37 °C. The mixtures were filtered through a PD G-25 size exclusion column (Cytiva, Marlborough, MA, USA) to remove any unloaded drugs. UV-Vis spectroscopy was used to measure the encapsulation efficiencies of T-Ce6 and PL. After the micelles were dissolved in dimethyl sulfoxide, the concentrations of the encapsulated PL and T-Ce6 were quantified by measuring their absorbance at 660 nm (for T-Ce6) and 328 nm (for PL), respectively. The standard curves of T-Ce6 and PL in DMSO were obtained in order to calculate their encapsulation efficiencies. The sizes and zeta potentials of T80(T-Ce6) and T80(T-Ce6/PL) were analyzed by dynamic light scattering (DLS) assessment using a Nano-ZS Zetasizer (Malvern, Worcestershire, UK) at 25 °C.

### 3.3. Assessment of Stability of T80(T-Ce6/PL)

To assess the colloidal stability of T80-based micelles when exposed to serum proteins, T80(T-Ce6/PL) was suspended in PBS containing 10% (*v*/*v*) FBS. Samples were incubated at 37 °C and then collected at different intervals (e.g., 24, 48, 72, and 96 h), and the size of T80(T-Ce6/PL) was measured by DLS (Nano-ZS Zetasizer, Malvern, Worcestershire, UK).

### 3.4. Drug Release Analysis 

The drug release profile of T80(T-Ce6/PL) micelles was obtained by incubating them in PBS (pH 7.4) at 37 °C for 48 h. T80 (49 µg) micelles containing 0.05 mg of T-Ce6 and 0.01 mg of PL were dispersed in PBS (100 µL) and loaded into a dialysis device (Slide-A-Lyzer Mini Dialysis, MW cutoff = 20 kDa, Thermo Fisher Scientific). The amounts of PL and T-Ce6 released from T80-based micelles at different time intervals were quantified by the measurement of their absorbance at 328 nm and 660 nm, respectively [10]. To determine the effect of US on the release of drugs from T80(T-Ce6/PL) micelles, samples were pretreated with 1 MHz US (0.3 W/cm^2^, 2 min) followed by incubation in the dark.

### 3.5. Cellular Uptake Analysis

Twelve-well plates were seeded with MCF-7 and hDFB cells (2 × 10^5^ cells/well) [11]. After overnight incubation, the cells received Ce6, T-Ce6, and T80(T-Ce6/PL) for 4 h at the equivalent concentration of Ce6 (2 µM). After trypsinization, the cells were rinsed with PBS. The fluorescence intensity of T-Ce6 taken up by the cells was quantified using a flow cytometer (CytoFLEX, Beckman Coulter, Brea, CA, USA).

### 3.6. Mitochondrial Uptake Evaluation 

Six-well plates were seeded with MCF-7 and hDFB cells (5 × 10^6^ cells/well) and incubated overnight. Then, they were incubated with Ce6, T-Ce6, and T80(T-Ce6/PL) at the equivalent concentration of Ce6 (or T-Ce6) (2 µM), followed by incubation for 4 h. After removing the media in the wells, we collected intact mitochondria using a Mitochondria/Cytosol Fractionation Kit (BioVision, San Francisco Bay, CA, USA). The mitochondrial uptake levels of Ce6 and T-Ce6 for each sample were assessed by flow cytometry (CytoFLEX).

For CLSM, MCF-7 cells (2 × 10^3^ cells/well) were seeded into a chambered coverglass (Lab-Tek, Thermo Fisher Scientific). After incubation overnight, cells were treated with Ce6, T-Ce6, and T80(T-Ce6/PL) at the equivalent concentration of Ce6 (or T-Ce6) (0.5 μM). After 2 h of incubation (37 °C), the cells were co-stained with MitoTracker Green (500 nM) and DAPI (500 nM). The cells in the glass-bottom dish were scanned using a confocal laser scanning microscope (Nikon C2 + DUVB, Nikon, Tokyo, Japan). The degree of colocalization between fluorescent signals was evaluated using Mander’s overlap coefficients. 

### 3.7. Intracellular ROS Quantification

Twenty-four-well plates were seeded with MCF-7 and hDFB cells (5 × 10^5^ cells/well) were seeded and incubated overnight. Then, the cells were treated with various samples, followed by incubation for 4 h. For US-treated groups, to minimize the thermal effect of US irradiation, the cells incubated with samples were exposed to US (1 MHz, 0.3 W/cm^2^, 2 min) at the bottom of the well plate with a layer of cold US gel. The cells were then stained with 10 µM of DCF-DA for 10 min. The fluorescence intensity emitted from the cells was measured by flow cytometry (CytoFLEX).

### 3.8. Cell Viability Analysis

A conventional MTT assay was performed for the assessment of anticancer activities of various samples. Briefly, 96-well plates were seeded with MCF-7 and hDFB cells (1 × 10^4^ cells/well) and incubated for 24 h. The cells in the wells received blank T80 micelles, Ce6, T-Ce6, T80(T-Ce6), and T80(T-Ce6/PL) (30 µg/mL of T80, 5 µM of Ce6 and T-Ce6, and 8 µM of PL). Untreated cells were adopted as a negative control. After an incubation of 4 h, the cells underwent US treatment (1 MHz, 0.3 W/cm^2^, 2 min), followed by an MTT assay [10].

### 3.9. Statistical Analysis 

Data were acquired in triplicate unless specified otherwise and expressed as mean ± standard deviation (SD) values. Statistical significance between sample groups was analyzed by one-way ANOVA and represented as follows: * *p* < 0.05; ** *p* < 0.01.

## 4. Conclusions

In summary, polysorbate-based micelles were developed to co-encapsulate mitochondria-specific sonosensitizers (T-Ce6) and pro-oxidant chemoagents (PL), enabling combined chemo-SDT. T80-based micelles markedly augmented the colloidal stability and cellular uptake of T-Ce6. US-responsive drug release was demonstrated using T80(T-Ce6/PL) micelles. T80(T-Ce6/PL) micelles generated high concentrations of intracellular ROS in MCF-7 human breast cancer cells upon exposure to US. Moreover, T80(T-Ce6/PL) demonstrated tumor-specific cytotoxicity owing to the high levels of oxidative stress in cancer cells. This combination of mitochondria-targeted SDT and pro-oxidant chemotherapy using polysorbate-based micelles holds significant promise for achieving effective and tumor-specific treatment. 

## Figures and Tables

**Table 1:** Sizes and zeta-potentials of various T80-based NPs.

	Blank T80	T80(T-Ce6)	T80(T-Ce6/PL)
Size (nm)	10.48 ± 0.46	18.03 ± 0.36	18.28 ± 8.49
Zeta potential (mV)	−18.4 ± 7.80	−9.36 ± 0.75	−9.08 ± 4.64
Polydispersity index (PDI)	0.059 ± 0.029	0.306 ± 0.083	0.381 ± 0.014

## Data Availability

All data presented in this study are included in the published article.
